# Percutaneous aspiration and sclerotherapy for simple hepatic cysts: a systematic review and meta-analysis

**DOI:** 10.1007/s11604-025-01874-7

**Published:** 2025-09-30

**Authors:** Tomohiro Matsumoto, Rika Yoshimatsu, Marina Osaki, Junki Shibata, Kana Miyatake, Tomoaki Yamanishi, Takuji Yamagami

**Affiliations:** 1 Department of Diagnostic and Interventional Radiology, Kochi Medical School, Kochi University, Oko-cho, Kohasu, Nankoku, Kochi 783-8505 Japan; 2 Department of Radiology, Kochi Health Sciences Center, 2125-1 Ike, Kochi, 781-0111 Japan

**Keywords:** Percutaneous aspiration and sclerotherapy, Simple hepatic cyst, Meta-analysis, Interventional radiology

## Abstract

**Purpose:**

This systematic review aims to assess the efficacy and safety of percutaneous aspiration and sclerotherapy (PAS) for patients with symptomatic simple hepatic cysts (SHCs).

**Materials and methods:**

We systematically searched the electronic databases of PubMed, Embase, the Cochrane Library and Ichushi-Web for studies published up to November 2024, reporting outcomes of PAS for symptomatic SHCs. The primary outcomes were rates of symptomatic relief or disappearance of symptoms. The secondary outcomes were cyst volume reduction rates and complication rates. Subgroup analyses compared ethanol with the other sclerosants. Single-arm meta-analyses were performed, with meta-regression conducted when substantial heterogeneity (*I*^2^ > 50%) was observed. Risk of bias was assessed using the Cochrane RoB2 tool for randomized controlled trials and RoBANS2 for non-randomized studies.

**Results:**

Sixteen studies were included. Fourteen studies were assessed as having a high risk of bias. The pooled symptomatic relief or disappearance rate was 86.9% (95% CI 80.2–91.6%, *I*^2^ = 0%). The cyst volume reduction rate was 86.4% (95% CI 74.1–93.3%, *I*^2^ = 95%). There were no major complications. The pooled minor complication rates were 13.6% (95% CI 6.5–26.4%, *I*^2^ = 67.2%) for pain and 7.4% (95% CI 4.1–13.0%, *I*^2^ = 38%) for fever. Subgroup analysis showed no significant differences between ethanol and other sclerosants. High heterogeneity was observed for cyst volume reduction and pain, indicating variability across studies. Meta-regression analysis for cyst volume reduction rate and pain did not identify any significant associations.

**Conclusion:**

PAS appears to be a relatively safe and effective treatment option for patients with symptomatic SHCs and provides high rates of symptomatic relief with low complication rates. However, given the high risk of bias in the available evidence and the lack of direct comparison with surgical treatment, these findings should be interpreted with caution. Further high-quality comparative studies are warranted to confirm these results.

**Supplementary Information:**

The online version supplementary material available at 10.1007/s11604-025-01874-7.

## Introduction

Simple hepatic cysts (SHCs), which are among the most commonly diagnosed benign liver lesions, are found in 18% of the general population following abdominal CT scans performed for unrelated conditions [1]. Although most SHCs are asymptomatic, they may become sufficiently large to cause symptoms such as abdominal distension, discomfort, and liver dysfunction [2, 3]. In such cases treatment is required, and there are several options available including percutaneous aspiration and sclerotherapy (PAS), and open or laparoscopic cyst deroofing [4]. Ethanol has long been the most commonly used sclerosant [5]; however, there has been increasing interest in other sclerosants such as minocycline, and tetracycline, which may have potentially fewer side effects or comparable efficacy [6–8]. Both surgical treatments versus PAS for symptomatic SHCs have been the subjects of a systematic review and meta-analysis [4]. To date, no dedicated systematic review and meta-analysis has focused exclusively on PAS for symptomatic SHCs, nor has there been a systematic analysis of how different sclerosants and procedural techniques may impact its efficacy. We aimed to fill this gap by systematically reviewing the available evidence on PAS using various sclerosants in patients with symptomatic SHCs.

## Materials and methods

We used guidelines from the Preferred Reporting Items for Systematic reviews and Meta-Analyses (PRISMA) to compile this report. The protocol was registered in PROSPERO (CRD42024585617).

### Search strategy

We systematically searched the electronic databases of the Cochrane Library, PubMed, Embase, and Ichushi-Web (Igaku Chuo Zasshi; Japan Medical Abstracts Society) for studies published up to November 2024. The literature search was carried out with the assistance of librarians (Supplementary Table S1).

### Inclusion and exclusion criteria

The inclusion criteria were as follows: (1) availability of full-text studies; (2) studies reporting data from PAS for patients with symptomatic SHCs (when studies also included other types of cysts—such as polycystic liver disease or mucinous cystic neoplasms—only patients with SHC were included); (3) studies of PAS for a solitary SHC and studies of PAS for only one cyst among multiple SHCs (in studies containing patients with multiple SHCs, only those patients for whom one cyst was treated were included); (4) studies published in English or Japanese. The exclusion criteria were as follows: (1) case reports; (2) review articles; (3) letters and editorials; (4) studies with a sample size of less than five cases; (5) studies with no extractable data; (6) a mean or median follow-up < 1 month; and (7) studies with data included in subsequent studies or duplicate reports.

### Outcomes and data extraction

The primary outcome was the clinical success rate, which was defined as symptomatic relief or disappearance of symptoms. The following secondary outcomes were also investigated: technical success rate (which was defined as procedure completion), major and minor complication rate, and cyst volume reduction rate and recurrence rate.

For each study, two reviewers (T. M. and J. S.) independently retrieved information on the outcomes and study characteristics, including the first author, publication year, study country, study design, and patient characteristics. For all studies, the following data were extracted separately for PAS for SHCs: clinical success, technical success, procedure details of PAS (including type of modality for puncture), whether a needle or catheter was used for sclerosant injection, aspiration volume, type and dosage of sclerosants, number of sessions (a pre-planned multi-procedure treatment was also defined as a single procedure), cyst volume reduction rate, sclerosant retention time, change in patient position, and sclerosant removal status. The complications were categorized in accordance with the classification system of the Cardiovascular and Interventional Radiological Society of Europe (CIRSE), i.e., from grade 1 (no complication) to grade 6 (death). Grades 3–6 were defined as major complications [9].

### Risk-of-bias assessment in the included studies

The quality of the included studies was assessed using version 2 of the Cochrane Collaboration tool for randomized controlled trials (RCTs) (RoB2) [10] and the revised Risk of Bias Assessment tool for Non-randomized Studies (RoBANS2) [11]. For the summary of risk of bias using RoBANS2, studies were considered to have an overall low risk of bias if they were classified as low risk in all eight domains. Studies were classified as having an unclear risk of bias if at least one domain was rated as unclear risk, provided that no domains were rated as high risk. Studies were considered to have a high risk of bias if at least one domain was rated as high risk.

### Subgroup analyses

Subgroup analyses were conducted where applicable to compare ethanol with other sclerosants.

### Statistical analyses

We performed single-arm meta-analyses, incorporating subgroup analysis and meta-regression models to control for confounding variables when comparing treatments. Where comparative studies were available, single-arm data were extracted from those studies and included in the meta-analysis. Considerable between-study heterogeneity was anticipated to impact the primary outcome; we therefore used a random-effects model to pool effect sizes based on the DerSimonian–Laird method. We also used Knapp–Hartung adjustments to calculate the confidence interval (CI) around the pooled effect. Forest plots were used to display outcome data, and the following heterogeneity measures were assessed: *τ*^2^, *I*^2^, and *Q*-statistics. For the *Q* statistic, a *p* value of < 0.05 was considered statistically significant. For the *I*^2^ statistic, values of 25% were defined as low heterogeneity, 25–50% were defined as moderate, and 50% were defined as high heterogeneity. Meta-regression analysis was conducted to identify the source of inter-study heterogeneity when *I*^2^ was greater than 50%. For meta-regression analysis, a value of *p* < 0.05 was set as the threshold for identification of the source of heterogeneity. Funnel plots and the Egger test were used to analyze publication bias; values of *p* < 0.05 were considered significant in the Egger test. The Egger test and meta-regression analysis were performed if at least four studies were selected for each meta-analysis. The same method was also used for analysis of secondary outcomes. For sensitivity analyses, we excluded studies with fewer than ten cases to reduce the impact of small case series, and studies published before 2000 to account for changes in sclerotherapy techniques and imaging guidance over time. Descriptive and basic statistics were run in Microsoft Excel (version16.94). The meta package of R software (version 4.4.0; R Foundation for Statistical Computing) was used for meta-analyses. Quality assessment plots were produced using risk-of-bias visualization ‘robvis’ [12].

## Results

### Search results

**Fig. 1:**
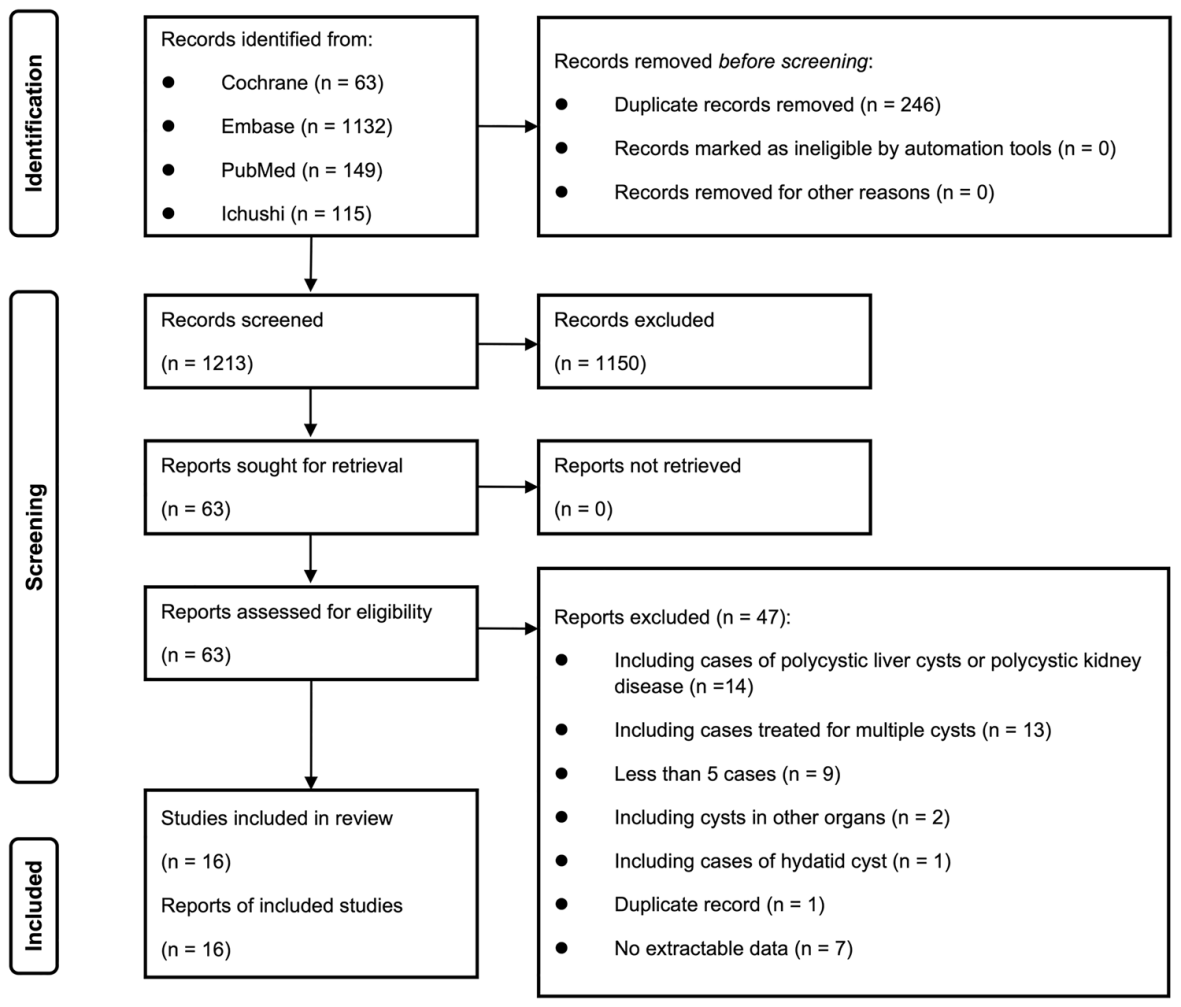
The preferred reporting items for systematic reviews and meta-analyses (PRISMA) flow diagram of the article selection process

Initial literature searches identified 1459 records, but elimination of duplicates yielded 1213 studies. Following the evaluation of titles and abstracts, 1150 studies were eliminated, leaving 63 of these records to undergo a full-text review. Finally, 47 studies were excluded by applying the eligibility criteria, leaving 16 studies for systematic review and meta-analysis of the available evidence on PAS using various sclerosing agents in patients with symptomatic SHCs [6–8, 13–25] (Fig. 1).

### Risk-of-bias assessment

**Fig. 2:**
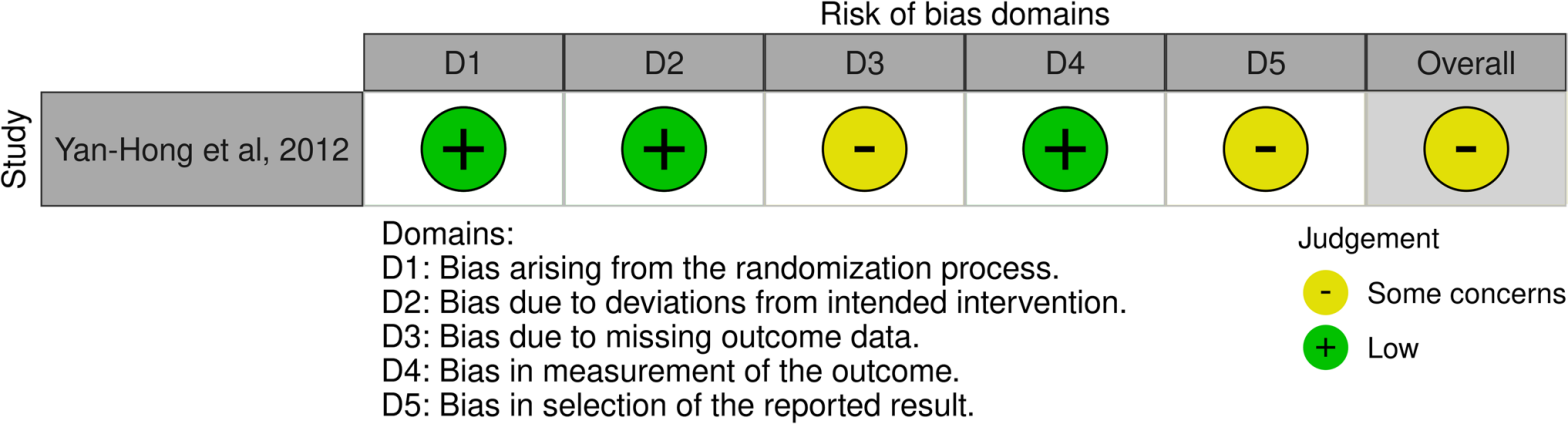
Risk-of-bias assessment using version 2 of the Cochrane Risk of Bias Tool for randomized controlled trials (RoB2). Green (+) indicates low risk of bias; yellow (-) indicates unclear risk of bias; and red (×) indicates high risk of bias

**Fig. 3:**
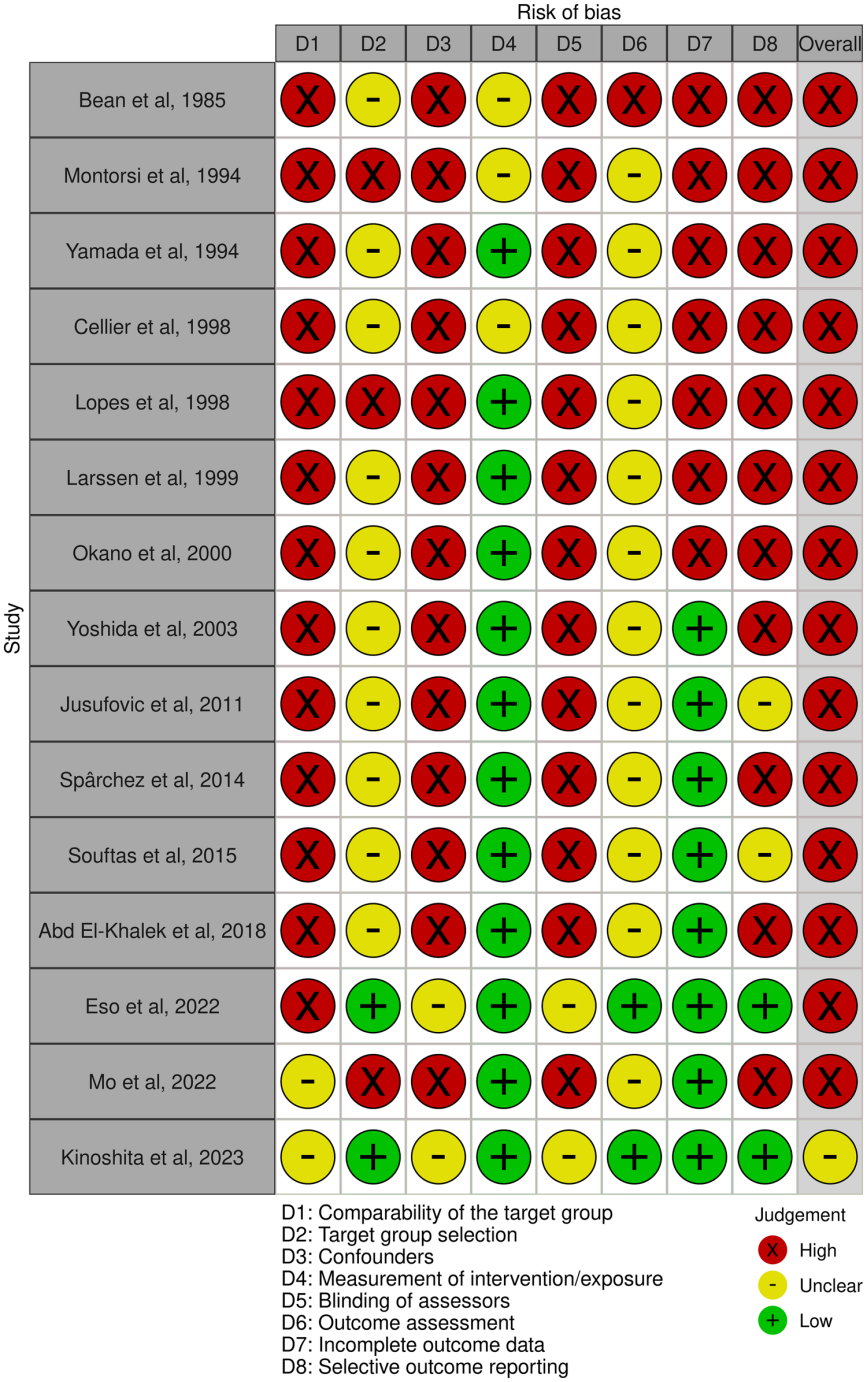
Risk-of-bias assessment using the revised Risk of Bias Assessment tool for Non-randomized Studies (RoBANS2). Green (+) indicates low risk of bias; yellow (-) indicates unclear risk of bias; and red (×) indicates high risk of bias. For the summary of risk of bias, studies were considered to have an overall low risk of bias (green +) if they were classified as low risk in all eight domains. Studies were considered to have an unclear risk of bias (yellow -) if at least one domain was rated as unclear risk (but no domains were rated as high risk) and as high risk (red ×) if at least one domain was rated as high risk

One study, which had reported RCT, was flagged by RoB2 as having some concerns (Fig. 2). Seven of the included studies were single-arm retrospective studies, four were single-arm prospective studies, two were retrospective cohort studies, and two were prospective cohort studies (Table 1). The bias of selected studies was assessed as high risk (*n* = 14) or unclear risk (*n* = 1) by RoBANS2 (Fig. 3).

**Table 1:** The characteristics of included articles for the present systematic review and meta-analysis

Author	Study type		Study period	Country	No. of patients	Male	Female	Mean age
Bean et al., 1985	R	Single arm	1982–1984	United States	5 of 6	2	3	63
Montorsi et al., 1994	R	Single arm	1987–1991	Italy	21 of 21	6	15	53
Yamada et al., 1994	R	Single arm	1990–1992	Japan	5 of 9	0	5	56.6
Cellier et al., 1998	R	Single arm	1992–1994	France	5 of 8	2	3	64.8
Lopes et al., 1998	R	Single arm	1993–1996	Portugal	7 of 7	2	5	52.6
Larssen et al., 1999	P	Single arm	1993–1998	Norway	11 of 11	1	10	65.3
Okano et al., 2000	R	Single arm	1986–1996	Japan	8 of 8	1	7	71.1
Yoshida et al., 2003	R	Single arm	1989–1998	Japan	9 of 9	2	7	58.2
Jusufovic et al., 2011	P	Single arm	NR	Bosnia and Herzegovina	20 of 20	7	13	52.9
Yan-Hong et al., 2012	RCT	Two arm	NR	China	33 of 33	27	40	61.8
					34 of 34			
Spârchez et al., 2014	P	Single arm	2008–2013	Romania	13 of 13	2	11	57.7
Souftas et al., 2015	P	Single arm	NR	Greece	7 of 14	1	6	62.7
Abd et al., 2018	P	Two arm Cohort	2013–2015	Egypt	18 of 70	Unknown	Unknown	Unknown
					16 of 70			
Eso et al., 2022	P	Two arm Cohort	2016–2021	Japan	15 of 15	2	13	65.3
Mo et al., 2022	R	Two arm Cohort	2013–2019	China	42 of 42	28	14	54.3
					39 of 39	23	16	54.6
Kinoshita et al., 2023	R	Two arm Cohort	2015–2019	Japan	7 of 11	2	5	72.7

*No. of patients* the number of patients extracted and included in this systematic review from each study, *NR* not reported, *P* prospective, *R* retrospective, *RCT* randomized controlled trial

### Characteristics of the included studies

The 16 selected studies involved a total of 315 cases (Table 1); these comprised 38% males and 62% females with an average-weighted mean age of 58.7 years. Among the 16 included studies, 7 were from Asia (Japan and China) and 9 were from other regions.

The details of the procedure are summarized in Table 2. Ultrasound guidance was used for cyst puncture in 13 studies [6–8, 13–20, 23, 25], while 2 studies performed puncture under CT guidance [21, 24]. In 5 studies, the sclerosant was injected using a needle [7, 8, 14, 16, 20], while in 11 studies, it was injected via a catheter [6, 13, 15, 17–19, 21–25]. Among the catheter group, pigtail catheters were commonly used [13, 17–19, 21, 23–25]. Ethanol was used in seven studies and was the most commonly studied sclerosant [6, 13, 15, 16, 19, 22, 24]. Other sclerosants investigated included minocycline chloride in four studies [7, 14, 17, 25], polidocanol in two studies [20, 23], tetracycline chloride in one study [8], 20% hypertonic saline solution in one study [18], OK-432 in one study [24], combination of 15% hypertonic saline and bleomycin in one study [21], and a combination of ethanol and tetracycline chloride in one study [22]. The injection volume of ethanol was set at 10–30% of the cyst volume or aspirated volume (*k* = 6) [6, 13, 15, 19, 22, 24] with a maximum dose of 100 mL (*k* = 4) [15, 16, 19, 22]. Among studies using ethanol as a sclerosant, the single-session injection method was the most used [5, 12, 14, 15, 18, 21]. In one study, repeated aspiration and instillation of ethanol was performed until the estimated alcohol concentration exceeded 80% [19]. The retention time of ethanol was 20 min in most studies (*k* = 5) [6, 16, 19, 22, 24]. The number of treatment sessions was most commonly reported as a single session (*k* = 5) [6, 13, 15, 19, 24]. Although the treatment was performed in a single session, one study adopted a two-step method in which ethanol was injected twice at a 12-h interval [24]. In one study, either one or two sessions were performed [16], and in another study, one, two, or three sessions were conducted [22]. Patient repositioning during ethanol injection was reported in four studies [6, 13, 16, 22], whereas the other three studies did not mention it [15, 19, 24]. Ethanol was aspirated after the procedure in all seven studies [6, 13, 15, 16, 19, 22, 24]. Minocycline hydrochloride was used in doses ranging from 100 to 500 mg. In two studies, the sclerosant was not aspirated [7, 14]. In two other studies, aspiration was not mentioned [17, 25]. Patient repositioning during both one and two sessions of minocycline hydrochloride injection was reported. Although the treatment was repeated daily for 7–8 days in one study [17], it was regarded as a single session in our analysis. There were reports of polidocanol being injected as a 3% solution [20] and as foam [23]. In one study using 3% polidocanol solution, 4–8 mL was injected per session and the sclerosant was not aspirated [20]. In one study using polidocanol foam (16 mL of 1% polidocanol per session), the catheter was left unclamped for open drainage until the next morning [23].

**Table 2:** Procedure details of percutaneous aspiration and sclerotherapy

Author	Modality for puncture	Devices used for sclerosant injection	Reduction rate	Type of Sclerosant	Treatment strategy	No. of sessions	Sclerosant retention time (min)	Patient position change	Sclerosant removal status
Bean et al., 1985	US	C (side hole)	NR	95% EtOH	< 400 mL: Inject 25% of the cyst volume > 400 mL: Two or more treatments at the same session	1	20	Yes	Yes
Montorsi et al., 1994	US	C (pigtail)	NR	95% EtOH	Inject 25% of the cyst volume	1	20–30	Yes	Yes
Yamada et al., 1994	US	N (21G)	NR	MINO	200 mg dissolved in 9 mL saline, mixed with 1 mL of 2% mepivacaine hydrochloride 100–600 mg (depending on its size) Repeated aspiration and injection of remaining cystic fluid including MINO several times	1, 2	NA	NR	No
Cellier et al., 1998	US	N (22G)	NR	MINO	100–500 mg dissolved in 10 mL saline 1 mg MINO per 1 mL cyst content Maximum dose: 500 mg 10 mL of 1% lidocaine into the cyst	1	NA	NR	No
Lopes et al., 1998	US	N (22G)	NR	TC	1000 mg dissolved in 10–80 mL saline	1	NA	NR	NR
Larssen et al., 1999	US	C (details unknown)	97.1 ± 6.1	96% EtOH	Inject 10% of the cyst volume Maximum dose: 100 mL	1	Up to 20	NR	Yes
Okano et al., 2000	US	N (21G)	NR	99% EtOH	20–100 mL	1, 2	20	Yes	Yes
Yoshida et al., 2003	US	C (pigtail)	NR	MINO	200 mg dissolved in 10 mL saline Flushed with 10 mL of saline Catheter clamped for 30 min Procedure Repeated daily for 7–8 days	1	NA	NR	NR
Jusufovic et al., 2011	US	C (pigtail)	96.3	20% HS	Inject 30% of the aspirated volume Maximum dose: 100 mL	1	120	Yes	Yes
Yan-Hong et al., 2012	US	C (pigtail)	93.6 ± 11.8	99% EtOH	Infused 10–15 mL 1% lidocaine 20–30% of the volume of the aspirated fluid Maximum dose: 100 mL	1	20	NR	Yes
		C (pigtail)	98.9 ± 1.8	99% EtOH	Infused 10–15 mL 1% lidocaine Repeated aspiration and instillation of alcohol until the estimated alcohol concentration exceeded 80%	1	NR	NR	Yes
Spârchez et al., 2014	US	N (18G)	66.3 ± 39.0	3% PLD	Inject 2–4 V (4–8 mL)	1, 2	NA	NR	No
Souftas et al., 2015	CT	C (pigtail)	90.3 ± 14.5	15% HS and BLM	Drained by gravity for 24 h Two injections and reabsorptions of 15% NaCl (20–25% of the cyst volume) Three times repetition of the same procedure with the addition of bleomycin hydrochloride (100 mg/m^2^)	1	3–5	NR	Yes
Abd et al., 2018	NR	C (details unknown)	56.7 ± 6.9	95% EtOH	Inject 20–25% of the aspirated fluid Maximum dose: 100 mL	1, 2, 3	20	Yes	Yes
		C (details unknown)	78.6 ± 8.5	95% EtOH and TC	Lower amount of alcohol Mixed with saline in 10 mL and injected without reaspiration	1,2	NR	Yes	NA
Eso et al., 2022	US	C (pigtail)	NR	PLD foam	4 mL of 1% polidocanol with 8–12 mL of CO_2_ 16 mL of 1% polidocanol per session Catheter was clamped for 15–20 min after the foam sclerosant injection Catheter unclamped for open drainage until the next morning	1, 2, 3	NA	Yes	NA
Mo et al., 2022	CT	C (pigtail)	NR	OK-432	0.1 mg of OK-432 dissolved in 10 mL of 0.9% saline 0.1 mg OK-432 per 10 mL cyst content Maximum dose: 100 mL	1	NA	NR	No
		C (pigtail)	NR	99% EtOH	Inject 25% of the aspirated fluid Performed twice with an interval of 12 h Left open for natural drainage between the injections Maximum single dose: 50 mL	1	20	NR	Yes
Kinoshita et al., 2023	US	C (pigtail)	94.6 ± 10.9	MINO	Inject 500 mg of MINO dissolved in 20 mL of saline	1, 2	NR	NR	NR

*BLM* bleomycin, *C* catheter, *EtOH* ethanol, *G* gauge, *HS* hypertonic saline, *MINO* minocycline hydrochloride, *N* needle, *NA* not applicable, *NR* not reported, *PLD* polidocanol, *TC* tetracycline chloride

### Analysis of the primary and secondary outcomes

**Fig. 4:**
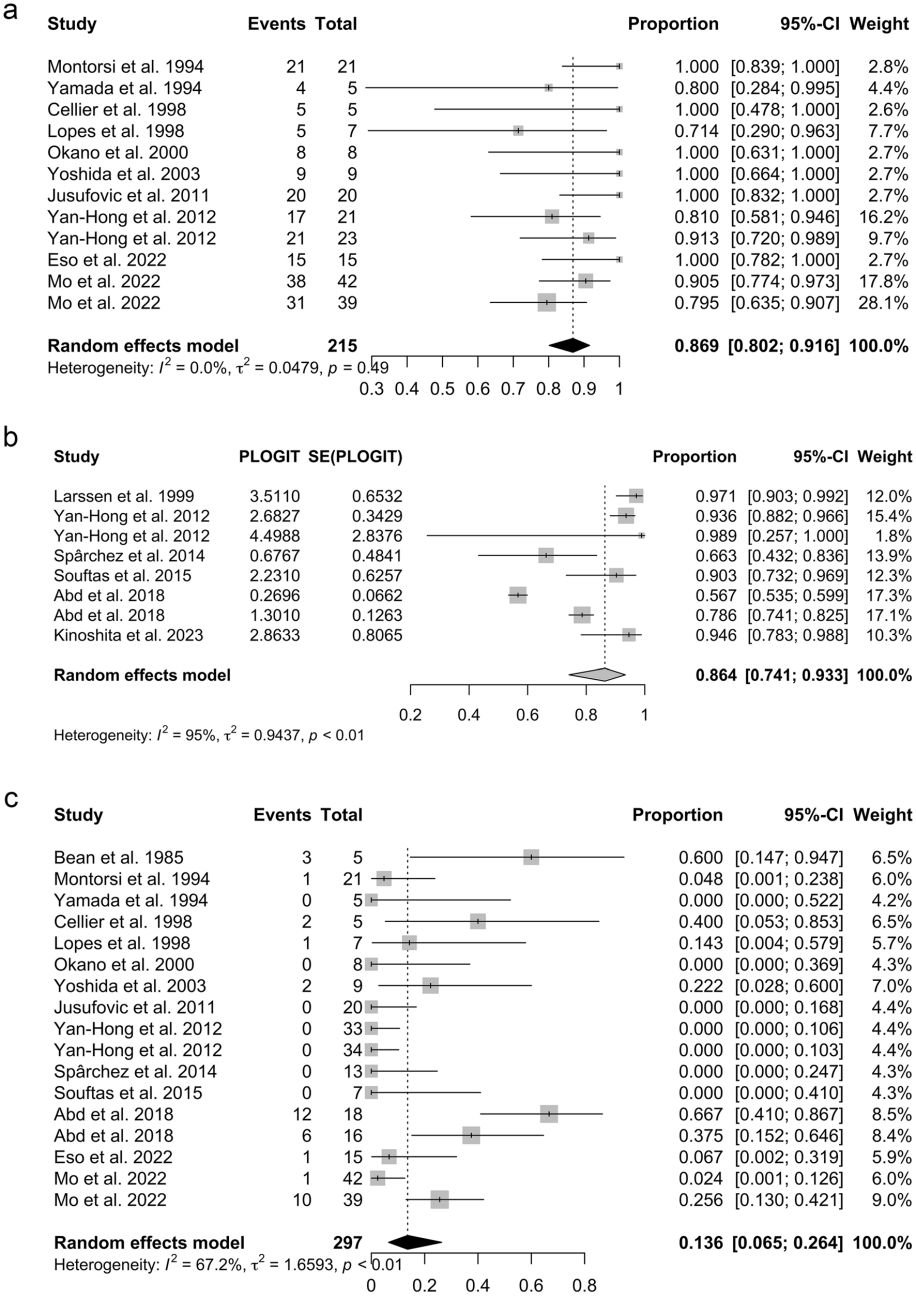

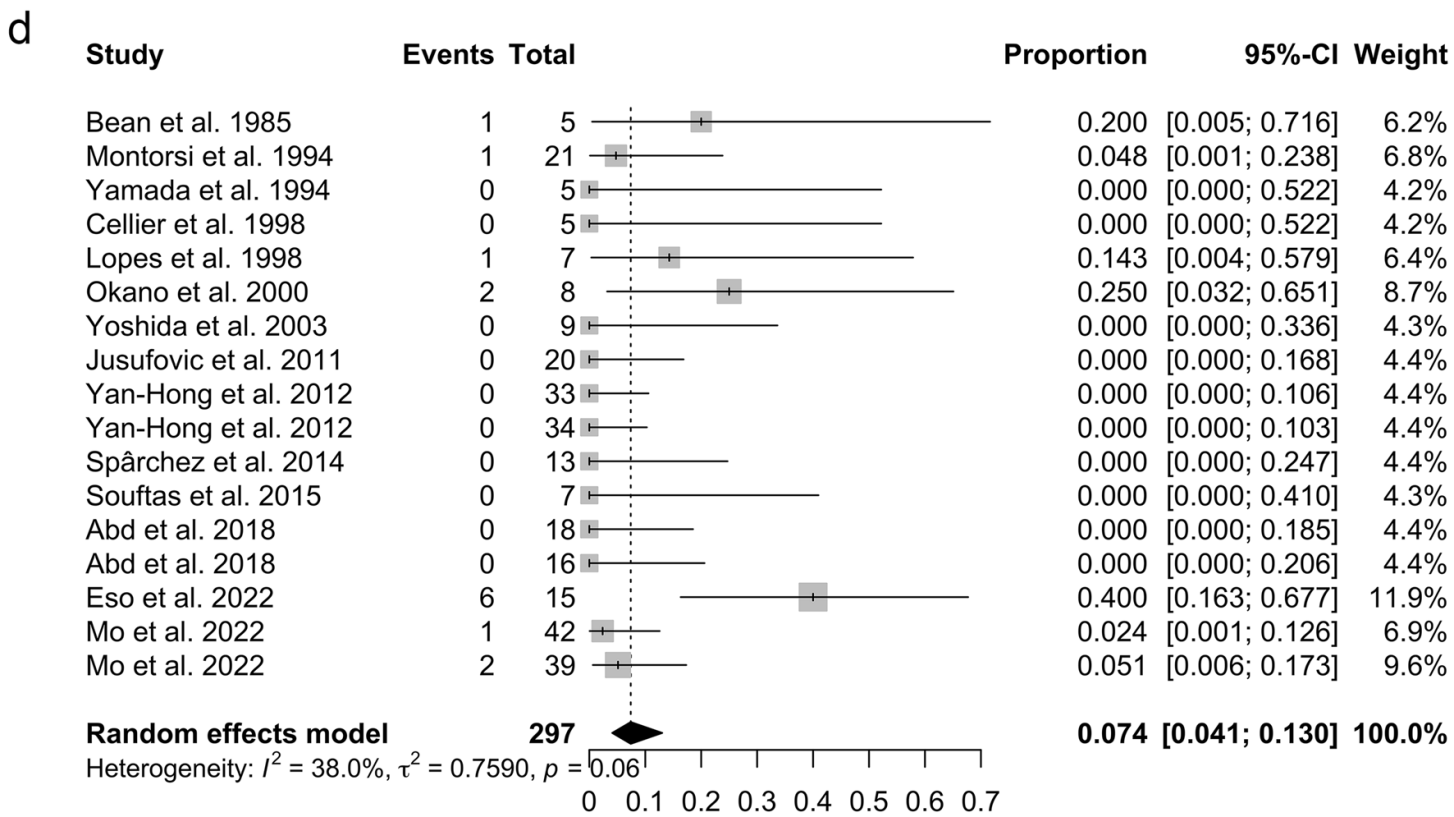
Forest plots of primary and secondary outcomes of percutaneous aspiration and sclerotherapy (PAS) for simple hepatic cysts (SHCs). **a** The forest plot of the pooled clinical success rates of PAS for SHCs. **b** The forest plot of the pooled cyst volume reduction rate of PAS for SHCs. **c** The forest plot of the pooled pain rate of PAS for SHCs. **d** The forest plot of the pooled fever rate of PAS for SHCs

**Fig. 5:**
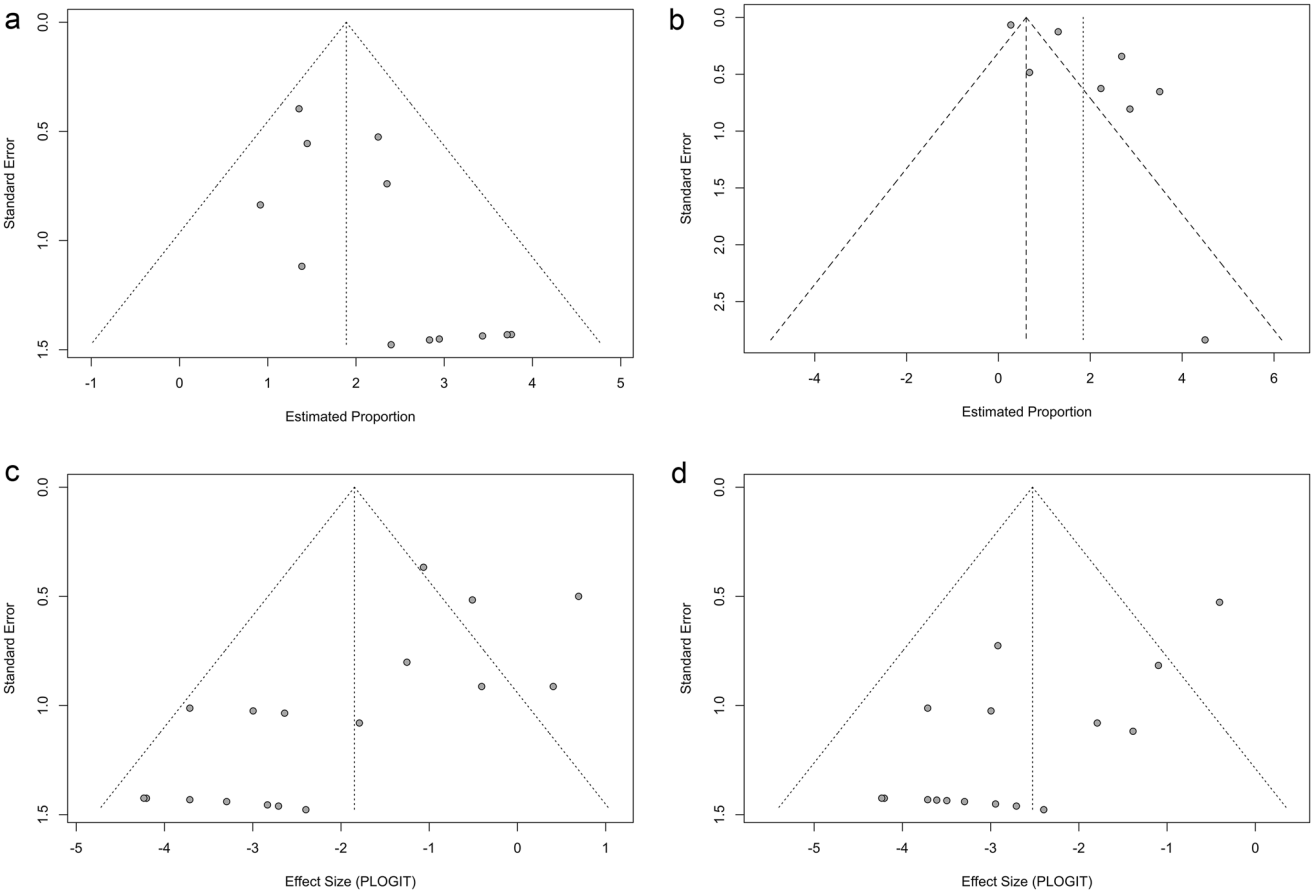
Funnel plots of the primary and secondary outcomes of percutaneous aspiration and sclerotherapy (PAS) for simple hepatic cysts (SHCs). **a** The funnel plot of the pooled clinical success rates of PAS for SHCs. **b** The funnel plot of the pooled cyst volume reduction rate of PAS for SHCs. **c** The funnel plot of the pooled pain rate of PAS for SHCs. **d** The funnel plot of the pooled fever rate of PAS for SHCs

The pooled clinical success rate was 86.9% (95% CI 80.2–91.6%, *I*^2^ = 0%, *p* = 0.49) (Fig. 4a). There was a significant publication bias for the pooled clinical success rate when assessed by Egger’s test (*p* = 0.017) and the funnel plot (Fig. 5a). The technical success rate was 100% in the included studies. The pooled cyst volume reduction rate was 86.4% (95% CI 74.1–93.3%, *I*^2^ = 95%, *p* < 0.01) (Fig. 4b). There was a significant publication bias for the pooled cyst volume reduction rate when assessed by Egger’s test (*p* = 0.037) and the funnel plot (Fig. 5b). Meta-regression analysis of the pooled cyst volume reduction rate revealed no significant association with sample size, publication year, or study region (Asia vs. other regions) (Table 3). The overall pooled rate of pain and fever classified as a minor complication was 13.6% (95% CI 6.5–26.4%, *I*^2^ = 67.2%, *p* < 0.01) (Fig. 4c) and 7.4% (95% CI 4.1–13.0%, *I*^2^ = 38%, *p* = 0.06) (Fig. 4d), respectively. There was a significant publication bias for the overall pooled rate of pain and fever when assessed by Egger’s test (*p* = 0.002 and 0.0008, respectively) and the funnel plots (Fig. 5c, 5d). Meta-regression analysis of the pooled proportion of pain revealed no significant association with sample size, publication year, or study region (Asia vs. other regions) (Table 3). Alcohol intoxication as a minor complication was reported in 4 out of 18 patients (22%) in one of the included studies [22]. Nausea as a minor complication was reported in one out of 15 patients (6%) in one of the included studies [23]. There were no major complications in any of the studies, and recurrence (1 out of 7 cases at 3 months) was only observed in one study [8].

**Table 3:** Meta-regression analysis of volume reduction rate and pain rate

Variable	*R*^2^ (%)	95% CI	*p* value
*Volume reduction rate*			
Publication year	30.2	− 0.21 to 0.01	0.08
Sample size	0	− 0.09 to 0.10	0.85
Region (Asia or elsewhere)	31.1	− 3.00 to 0.12	0.07
*Pain*			
Publication year	0	− 0.09 to 0.05	0.56
Sample size	2.7	− 0.1 to 0.02	0.15
Region (Asia or elsewhere)	18.4	− 0.3 to 2.9	0.09

*CI* confidence interval

### Subgroup analysis

Subgroup analysis comparing ethanol and other sclerosants for clinical success rate, cyst volume reduction rate, and rate of pain and fever as minor complications (Table 4) revealed no significant differences. However, substantial heterogeneity was observed for pain incidence in the ethanol subgroup (*I*^2^ = 83.6%), indicating variability in study results. Similarly, high heterogeneity was observed for cyst volume reduction rate in both the ethanol group (*I*^2^ = 95.9%) and the other sclerosant group (*I*^2^ = 60.3%).

**Table 4:** Subgroup analysis of PAS for SHC

Outcome	Rate (%)	95% CI	*I*^2^ (%)	*p* for subgroup analysis
*Clinical success rate (k = 12)*				0.32
Ethanol (*k* = 5)	84.3	70.0–92.5	9.4	
Other sclerosants (*k* = 7)	89.4	79.1–95.0	0	
*Volume reduction rate (k = 8)*				0.48
Ethanol (*k* = 4)	91.0	57.6–98.7	95.9	
Other sclerosants (*k* = 4)	82.4	69.6–90.5	60.3	
*Rate of pain (k = 17)*				0.37
Ethanol (*k* = 7)	9.9	1.5–45.1	83.6	
Other sclerosants (*k* = 10)	19.6	10.6–33.3	23.7	
*Rate of fever (k = 17)*				0.45
Ethanol (*k* = 7)	5.7	1.7–17.3	33.2	
Other sclerosants (*k* = 10)	8.9	4.1–18.2	39.4	

*CI* confidence interval, *PAS* percutaneous aspiration and sclerotherapy, *SHC* simple hepatic cyst

### Sensitivity analyses

The results of the sensitivity analyses largely overlapped with those of the primary analyses, showing similar outcomes and indicating the robustness of the findings (Supplementary Table S2).

## Discussions

This systematic review and meta-analysis evaluated the efficacy and safety of PAS for symptomatic SHCs and examined differences between ethanol and other sclerosants in subgroup analyses. Our findings provide insights into PAS for symptomatic SHCs and may serve as a valuable reference for clinical decision-making. However, the pooled clinical success rate in our meta-analysis was lower than that reported by Furumaya et al. [4]. We believe that this discrepancy may be explained by differences in the meta-analysis methodology, including our use of the DerSimonian–Laird random-effects model with Knapp–Hartung adjustment instead of the Freeman–Tukey double-arcsine transformation. In addition, we included several recently published studies (e.g., Mo et al. [24]) with relatively lower success rates but larger sample sizes, which contributed higher weights to the pooled estimate. Although further research is warranted, PAS appears to be a relatively safe and effective treatment option for symptomatic SHCs. In line with Furumaya et al. [4], a step-up approach, in which surgical interventions such as laparoscopic cyst deroofing are reserved for cases of recurrence after PAS, may be considered; however this needs confirmation through comparative studies.

The most studied sclerosant in this systematic review was ethanol, which destroys the epithelial lining of the cystic cavity, thereby inhibiting cystic fluid secretion [6]. Ethanol was most often introduced via the single-session injection method, as it is simple and can be completed quickly [6, 13, 15, 16, 19, 22]. The ethanol retention time was generally around 20–30 min [6, 15, 16, 19, 22], and some studies also mentioned patient repositioning during the procedure [6, 13, 16, 22]. Therefore, the single-session injection method using ethanol is a relatively robust technical procedure. None of the included studies reported major complications, either in the ethanol group or in other sclerosant group, suggesting a favorable safety profile for both approaches. Ethanol, however, may leak from the cystic cavity into the peritoneum or bile ducts and be systemically absorbed, potentially leading to hypotension, liver abscess, pleuritis, cholangitis, and intracystic hemorrhage [26]. Subgroup analysis revealed no significant differences in the clinical success rate (*p* = 0.32) and volume reduction rate (*p* = 0.48) between ethanol and other sclerosants. For clinical success, heterogeneity was low in the ethanol group (*I*^2^ = 9.4%) and absent in the other sclerosant group (*I*^2^ = 0%), suggesting that the pooled estimates were relatively consistent across studies. In contrast, volume reduction rate showed considerable heterogeneity in both groups: *I*^2^ = 95.9% in the ethanol group and *I*^2^ = 60.3% in the other sclerosant group. In the ethanol group, treatment protocols included not only the single-session injection method, but also other techniques, while the number of studies on alternative sclerosants was limited and procedural techniques varied widely across those studies. Therefore, the subgroup results for volume reduction rates should be interpreted with caution. Moreover, subgroup analysis revealed no significant differences in minor complications, such as pain (*p* = 0.37) and fever (*p* = 0.45), between ethanol and other sclerosants. However, the incidence of pain in the ethanol group showed substantial heterogeneity (*I*^2^ = 83.6%), likely due to differences in pain assessment criteria across studies. Thus, careful interpretation of these findings is warranted. Despite variability in pain incidence and volume reduction rates, ethanol remains the most technically stable and widely studied sclerosant for PAS in the treatment of symptomatic SHCs. However, alternative sclerosants could also be effective, given our findings that they elicited similar symptom relief rates and cyst volume reduction rates as ethanol. Further studies are needed to better evaluate the efficacy and safety of these alternative sclerosants.

Due to insufficient data on mean age and sex ratio in some studies, meta-regression analyses could not be performed for these factors in relation to cyst volume reduction and pain. Although we explored potential regional heterogeneity through meta-regression, the studies from Asia were limited to Japan and China, which may limit the generalizability of our findings across the entire Asian region. Furthermore, even after performing a meta-regression analysis using the available data, no significant factors were identified to explain the heterogeneity in cyst volume reduction and pain. This suggests that the clinical outcomes of PAS may be influenced by factors beyond these common variables, which were not examined in this study. In particular, treatment strategies varied among the included studies [15, 19–22, 25], potentially affecting the assessment of cyst volume reduction. Therefore, standardizing future treatment strategies will be essential to ensure a more accurate evaluation of PAS outcomes.

Significant publication bias was observed across all outcomes in this study. This bias may be attributed to the tendency for positive results to be published, while negative results are often rejected or not even submitted. This limitation highlights the need for a more balanced and comprehensive evidence base. Additionally, the limited number of comparative studies and the predominance of retrospective designs may have introduced further biases, potentially affecting the reliability of the findings. Future high-quality prospective studies with standardized evaluation criteria are needed to confirm these results and further assess the efficacy and safety of different sclerosants.

This study has several limitations. First, our systematic review and meta-analysis focused on symptomatic relief or the disappearance of symptoms as the primary outcome, which is a subjective measure. Ideally, assessments should be conducted using validated tools such as the visual analog scale (VAS), quality of life (QOL) scores, or symptom evaluation scales. However, none of the included studies utilized these measures for evaluation. Future studies are expected to incorporate these standardized measures. Second, most of the included studies had a retrospective design and a high overall risk of bias. Third, only five comparative studies were identified, limiting the ability to conduct comparative meta-analyses. Consequently, this study focused on obtaining single-arm pooled estimates and performing subgroup analyses. Given the predominance of single-arm studies, further prospective comparative studies are warranted to confirm these findings. Despite these limitations, this systematic review and meta-analysis provides valuable insights into the current clinical utility of PAS for symptomatic SHCs.

In conclusion, our study suggests that PAS appears to be a relatively safe and effective treatment option for patients with symptomatic SHCs and provides high rates of symptomatic relief with low complication rates.

## Supplementary Information

Below is the link to the electronic supplementary material.

Supplementary material 1 (DOCX 32 KB)
