# Supplementary material for: Percutaneous aspiration and sclerotherapy for simple hepatic cysts: a systematic review and meta-analysis
Source: Jpn J Radiol. 2025 Sep 30;44(1):168–82. doi: 10.1007/s11604-025-01874-7 (PMC12769637; doi:10.1007/s11604-025-01874-7)
Supplement: Supplementary file 1 — Supplementary material 1 (DOCX 32 KB) [file 11604_2025_1874_MOESM1_ESM.docx]

**Percutaneous aspiration and sclerotherapy for simple hepatic cysts: a systematic review and meta-analysis**

Japanese Journal of Radiology

**Authors:** Tomohiro Matsumoto^1)^, Rika Yoshimatsu^1, 2)^, Marina Osaki^1)^, Junki Shibata^1)^, Kana Miyatake^1)^, Tomoaki Yamanishi^1)^, Takuji Yamagami^1)^

**Affiliations:**

1. Department of Diagnostic and Interventional Radiology, Kochi Medical School, Kochi University, Oko-cho, Kohasu, Nankoku, Kochi, 783-8505, Japan
2. Department of Radiology, Kochi Health Sciences Center, 2125-1 Ike, Kochi, 781-0111, Japan

* Correspondence to Associate Professor Tomohiro Matsumoto, M.D., Ph.D.

Associate Professor, Department of Diagnostic and Interventional Radiology, Kochi University, Kochi Medical School, Kohasu, Oko-cho, Nankoku, Kochi 783-8505, Japan.

(ORCID ID https://orcid.org/0000-0002-2375-4919)

Tel: +81 888802367

FAX: +81 888802368

E-mail: [t-matsu@kochi-u.ac.jp](mailto:t-matsu@kochi-u.ac.jp)

Supplementary Table S1. Literature search.

1. Library Search Strategy: Cochrane Library.

| Search No | Search Strategy |
| --- | --- |
| #01 | ((liver* OR hepatic*) AND cyst*):ti,ab,kw |
| #02 | (sclerot* OR "Sclerosing Solutions"):ti,ab,kw |
| #03 | (aspirat* OR drainage*):ti,ab,kw |
| #04 | (inject* OR infus*):ti,ab,kw |
| #05 | #1 AND (#2 OR (#3 AND #4)) |
| #06 | #5 CDSR |
| #07 | #5 CCRCT |

1. Library Search Strategy: PubMed.

| Search No | Search Strategy |
| --- | --- |
| #01 | Liver[Mesh] OR "Liver Diseases"[Mesh] OR liver*[tiab] OR hepatic[tiab] |
| #02 | Cysts[Mesh] OR "Cyst Fluid"[Mesh] OR cyst*[tiab] |
| #03 | #1 AND #2 |
| #04 | Sclerotherapy[Mesh] OR sclerot*[tiab] OR "Sclerosing Solutions"[Mesh] OR "Sclerosing Solutions" [Pharmacological Action] OR "Sclerosing Solution*"[tiab] |
| #05 | aspirat*[tiab] OR "Drainage"[Mesh] OR drainage*[tiab] |
| #06 | "Injections, Intralesional"[Mesh] OR inject*[tiab] OR "Infusions, Intralesional"[Mesh] OR infus*[tiab] |
| #07 | #4 OR (#5 AND #6) |
| #08 | #3 AND #7 |
| #09 | #8 AND ("Meta-Analysis"[PT] OR "Meta-Analysis as Topic"[Mesh] OR "meta-analysis"[TIAB]) |
| #10 | #8 AND ("Cochrane Database Syst Rev"[TA] OR "Systematic Review"[PT] OR "Systematic Reviews as Topic"[Mesh] OR "systematic review"[TIAB]) |
| #11 | #8 AND ("Practice Guideline"[PT] OR "Practice Guidelines as Topic"[Mesh] OR "Consensus"[Mesh] OR "Consensus Development Conferences as Topic"[Mesh] OR "Consensus Development Conference"[PT] OR guideline*[TI] OR consensus[TI]) |
| #12 | #9 OR #10 OR #11 |
| #13 | #8 AND ("Randomized Controlled Trial"[PT] OR "Randomized Controlled Trials as Topic"[Mesh] OR (random*[TIAB] NOT medline[SB])) |
| #14 | #8 AND ("Clinical Trial"[PT] OR "Clinical Trials as Topic"[Mesh] OR "Observational Study"[PT] OR "Observational Studies as Topic"[Mesh] OR ((clinical trial*[TIAB] OR case control*[TIAB] OR case comparison*[TIAB]) NOT medline[SB])) |
| #15 | (#13 OR #14) NOT #12 |
| #16 | #8 AND ("Epidemiologic Methods"[Mesh] OR "Comparative Study"[PT] OR "Multicenter Study"[PT] OR "Validation Study"[PT] OR (("survival analysis"[TIAB] OR cohort*[TIAB] OR comparative stud*[TIAB] OR follow-up stud*[TIAB] OR prospective stud*[TIAB] OR Retrospective study*[TIAB] OR sensitivit*[TIAB] OR specificit*[TIAB]) NOT medline[SB])) |
| #17 | #16 NOT (#12 OR #15) |
|  |  |

1. Library Search Strategy: Embase.

| No | 項目 |
| --- | --- |
| #1 | liver'/exp OR 'liver disease'/exp OR 'liver*':ti,ab,kw OR 'hepatic':ti,ab,kw |
| #2 | cyst'/exp OR 'cyst*':ti,ab,kw |
| #3 | #1 AND #2 |
| #4 | sclerotherapy'/exp OR 'sclerot*':ti,ab,kw OR 'sclerosing agent'/exp OR 'sclerosing agent*':ti,ab,kw OR 'sclerosing solution*':ti,ab,kw |
| #5 | aspiration, puncture and suction'/exp OR 'aspirat*':ti,ab,kw OR 'percutaneous drainage'/exp OR 'drainage*':ti,ab,kw |
| #6 | inject*':ti,ab,kw OR 'intralesional drug administration'/exp OR 'infus*':ti,ab,kw OR 'drug infusion'/exp |
| #7 | #4 OR (#5 AND #6) |
| #8 | #3 AND #7 |

1. Library Search Strategy: Ichushi-Web.

| Search No | Search Strategy |
| --- | --- |
| #01 | 肝臓/TH or 肝臓疾患/TH or 肝臓/TA or 肝臓疾患/TA or 肝疾患/TA or 肝障害/TA or 肝病変/TA |
| #02 | 嚢胞/TH or Cyst/TA or のう胞/TA or シスト/TA or 嚢腫/TA or 包嚢/TA |
| #03 | #1 and #2 |
| #04 | 硬化療法/TH or 硬化療法/TA or Sclerot/TA or 硬化剤/TH or 硬化剤/TA |
| #05 | ドレナージ/TH or ドレナージ/TA or drainage/TA or 吸引/TA or aspirat/TA |
| #06 | 病巣内投与/TH or 注入/TA or inject/TA or infus/TA |
| #07 | #4 or (#5 and #6) |
| #08 | #3 and #7 |
| #09 | #8 and (メタアナリシス/TH or システマティックレビュー/TH) |
| #10 | #8 and (RD=メタアナリシス) |
| #11 | #8 and (メタアナリシス/TA or システマティックレビュー/TA) |
| #12 | #8 and 診療ガイドライン/TH |
| #13 | #8 and (RD=診療ガイドライン) |
| #14 | #8 and 診療ガイドライン/TA |
| #15 | #9 or #10 or #11 or #12 or #13 or #14 |
| #16 | #8 and ランダム化比較試験/TH |
| #17 | #8 and (RD=ランダム化比較試験) |
| #18 | #8 and (ランダム化/TA or 無作為化/TA) |
| #19 | #8 and (疫学研究特性/TH or 疫学的研究デザイン/TH) |
| #20 | #8 and (RD=準ランダム化比較試験,比較研究) |
| #21 | #8 and (疫学研究/TA or 疫学的研究/TA or 観察研究/TA or 縦断研究/TA or 後向き研究/TA or 症例対照研究/TA or 前向き研究/TA or コホート研究/TA or 追跡研究/TA or 断面研究/TA or 介入研究/TA or 実現可能性研究/TA or 双生児研究/TA or 多施設共同研究/TA or パイロットプロジェクト/TA or 標本調査/TA or 臨床試験/TA or 第I相試験/TA or 第II相試験/TA or 第III相試験/TA or 第IV相試験/TA or 一重盲検法/TA or クロスオーバー研究/TA or 結果再現性/TA or 寸法測定精度/TA or 対照群/TA or 追跡不能例/TA or 二重盲検法/TA or 標本サイズ/TA or マッチドペア分析/TA) |
| #22 | (#16 or #17 or #18 or #19 or #20 or #21) not #15 |
| #23 | #8 and (PT=原著論文,総説) |
| #24 | #23 not (#15 or #22) |

| Supplementary Table S2. Comparison of primary analyses and sensitivity analyses of PAS for SHC. | | |
| --- | --- | --- |
| Clinical success rate | Rate (%) | 95% CI |
| All studies | 86.9 | 80.2–91.6 |
| Studies published in or after 2000 with at least 10 cases | 86.8 | 75.7–93.2 |
| Cyst volume reduction rate |  |  |
| All studies | 86.4 | 74.1–93.3 |
| Studies published in or after 2000 with at least 10 cases | 78.5 | 60.0–89.4 |
| Pain |  |  |
| All studies | 13.6 | 6.5–26.4 |
| Studies published in or after 2000 with at least 10 cases | 10.5 | 2.6–31.8 |
| Fever |  |  |
| All studies | 7.4 | 4.1–13.0 |
| Studies published in or after 2000 with at least 10 cases | 4.7 | 1.7–12.4 |
| CI, confidence interval; PAS, percutaneous aspiration and sclerotherapy; SHC, simple hepatic cyst. | | |
